# Commentary: Research Gaps in Gestational Diabetes Mellitus: Executive Summary of a National Institute of Diabetes and Digestive and Kidney Diseases Workshop

**DOI:** 10.3389/fendo.2018.00627

**Published:** 2018-10-19

**Authors:** Andrew A. Bremer

**Affiliations:** Pediatric Growth and Nutrition Branch, Eunice Kennedy Shriver National Institute of Child Health and Human Development, National Institutes of Health, Bethesda, MD, United States

**Keywords:** gestational diabetes, metabolic programming, hyperglycemia in pregnancy, maternal outcome, fetal outcome

Diabetes mellitus diagnosed in pregnancy, or gestational diabetes mellitus (GDM), is a problem of increasing public health importance in the United States and worldwide. GDM is associated with significantly increased risks of adverse perinatal outcomes as well as an increased risk of subsequent type 2 diabetes (T2D) in the mother, and obesity in offspring (1–3). Its prevalence is also increasing with the current obesity epidemic and advancing age of motherhood (1). Furthermore, based on the Hyperglycemia and Adverse Pregnancy Outcomes (HAPO) study, there appears to be a strong linear relationship between increasing maternal glucose concentrations (below those currently used to diagnose overt diabetes) and adverse neonatal outcomes without any clear inflection point (4). Data from the HAPO follow-up study at 8–12 years after the pregnancy will also soon provide further insight into the relationship between glucose levels below the current GDM diagnostic threshold and subsequent T2D in mothers and obesity in the offspring.

Importantly, many unanswered questions exist about the role of maternal glucose intolerance in fetal metabolic imprinting, and whether identifying and effectively treating maternal dysglycemia earlier in pregnancy than the current “standard of care” assessment of maternal dysglycemia at 24–28 weeks gestation would mitigate potential short- and long-term effects on the mother and/or offspring. Specifically, little is known about variations in maternal glycemia prior to 20 weeks gestation. As such, questions such as the following persist: (i) do glycemic measures (e.g., various degrees of maternal glucose intolerance as measured by oral glucose tolerance testing [OGTT], hemoglobin A1c [HbA1c], or continuous glucose monitoring [CGM]), or other biomarkers in early gestation predict GDM in later pregnancy and/or other adverse outcomes in the mother and/or the offspring; and (ii) would early diagnosis and treatment of maternal dysglycemia improve short- and long-term outcomes for the mother and her child?

To address these issues, the National Institute of Diabetes and Digestive and Kidney Diseases (NIDDK) at the National Institutes of Health convened an international workshop in August 2017 involving obstetricians, maternal-fetal medicine specialists, internists, and endocrinologists with expertise in GDM to address the current gaps in GDM research. Areas of particular focus included the lack of knowledge on when dysglycemia manifests during pregnancy and the potential importance of early diagnosis of GDM, and evidence for and against different treatment strategies and therapeutic goals in the management of GDM. As summarized in a recently published Executive Summary of the workshop (5), appropriate diagnostic criteria for the diagnosis of GDM unfortunately remain poorly defined, and an effect of early diagnosis and treatment of GDM on the risk of adverse perinatal and long-term outcomes has not been well demonstrated. Furthermore, despite many small randomized controlled trials of glucose-lowering medication treatment in GDM, gaps remain in understanding how best to utilize pharmacological therapy in GDM, as evidenced by discrepancies among professional society treatment guidelines. The comparative effectiveness of insulin, metformin, and glyburide in the management of GDM also remains uncertain, particularly with respect to long-term maternal and fetal outcomes. The NIDDK workshop participants also identified additional topics in need of further research, including phenotypic heterogeneity in GDM and novel and individualized treatment approaches. Interest in the topic of pregnancies complicated by GDM (as well as pre-existing diabetes) is also highlighted by the American Diabetes Association including in its July 2018 issue of *Diabetes Care* a Special Article Collection entitled “Reconsidering Pregnancy With Diabetes.”

In summary, the many unanswered questions posed above highlight some, but certainly not all, of the knowledge gaps in the field of GDM as it pertains to the mother and her offspring. As the field moves forward, filling these gaps will better elucidate our understanding of the physiology of glucose homeostasis during pregnancy, as well as our understanding of pathophysiological changes and the timing and approach of medical management of maternal glucose intolerance to mitigate short- and long-term adverse effects on the mother and the offspring. Although advances in diabetes technologies (such as CGM) have the potential to facilitate these efforts (6), their large-scale use in women with or at-risk for GDM is limited at best. However, these technological advances provide opportunities for interrogating gestational glycemia—both untreated and in response to therapy—in ways that were previously undoable or impractical. As the American Diabetes Association so aptly alluded to on the cover of its July 2018 issue of *Diabetes Care*, it is time to reconsider pregnancy with diabetes—in all facets.

## Author contributions

The author confirms being the sole contributor of this work and has approved it for publication.

### Conflict of interest statement

The author declares that the research was conducted in the absence of any commercial or financial relationships that could be construed as a potential conflict of interest.
